# Supplementary figures and images for: Bioengineered Skin from a Platelet-Derived Hydrogel Repairs Full Thickness Wounds in a Pre-Clinical Mouse Model
Source: Int J Mol Sci. 2025 Oct 14;26(20):9988. doi: 10.3390/ijms26209988 (PMC12564419; doi:10.3390/ijms26209988)

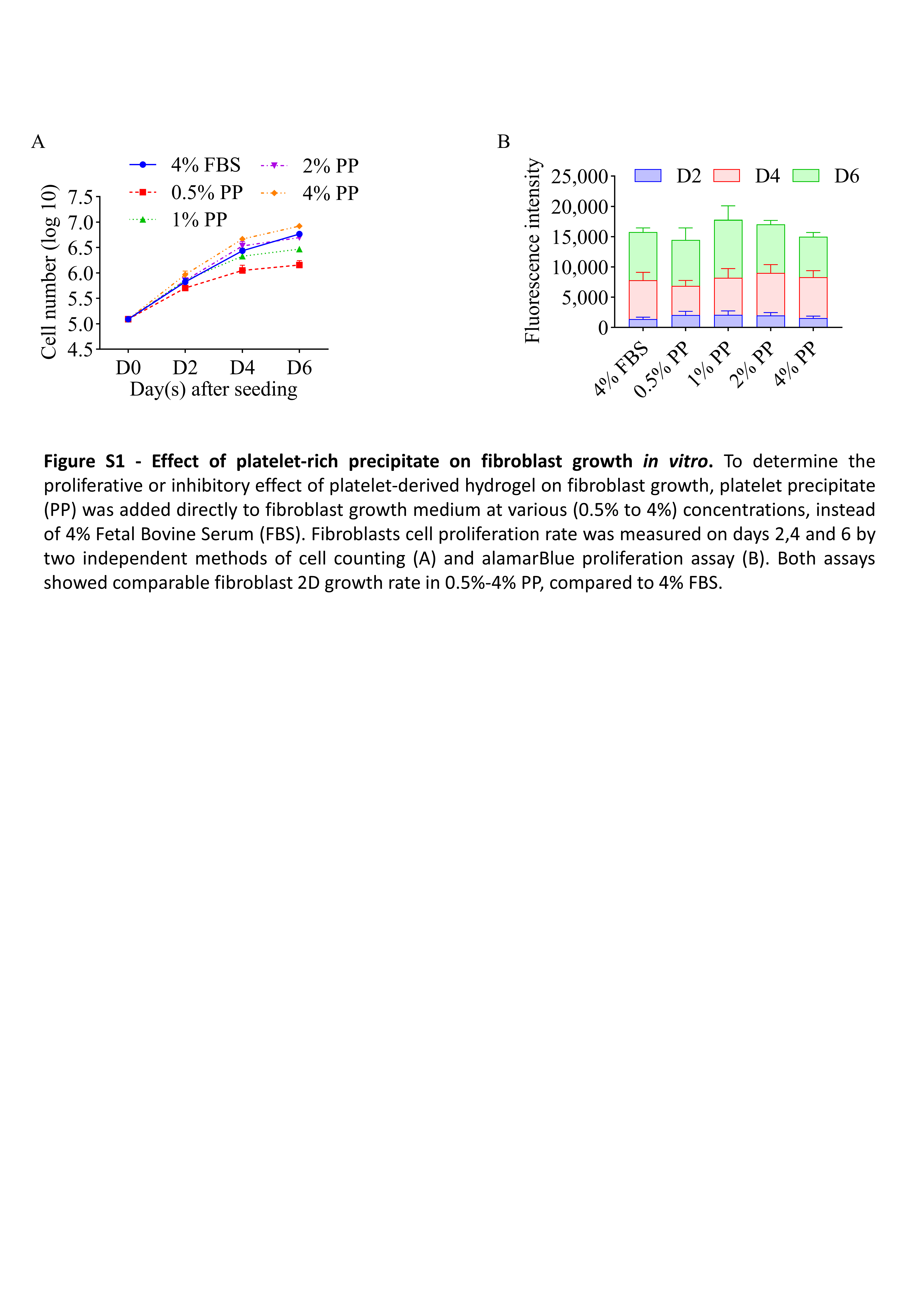

Supplement: Supplementary file 1 [file ijms-26-09988-s001.zip › Figure S1.TIF]

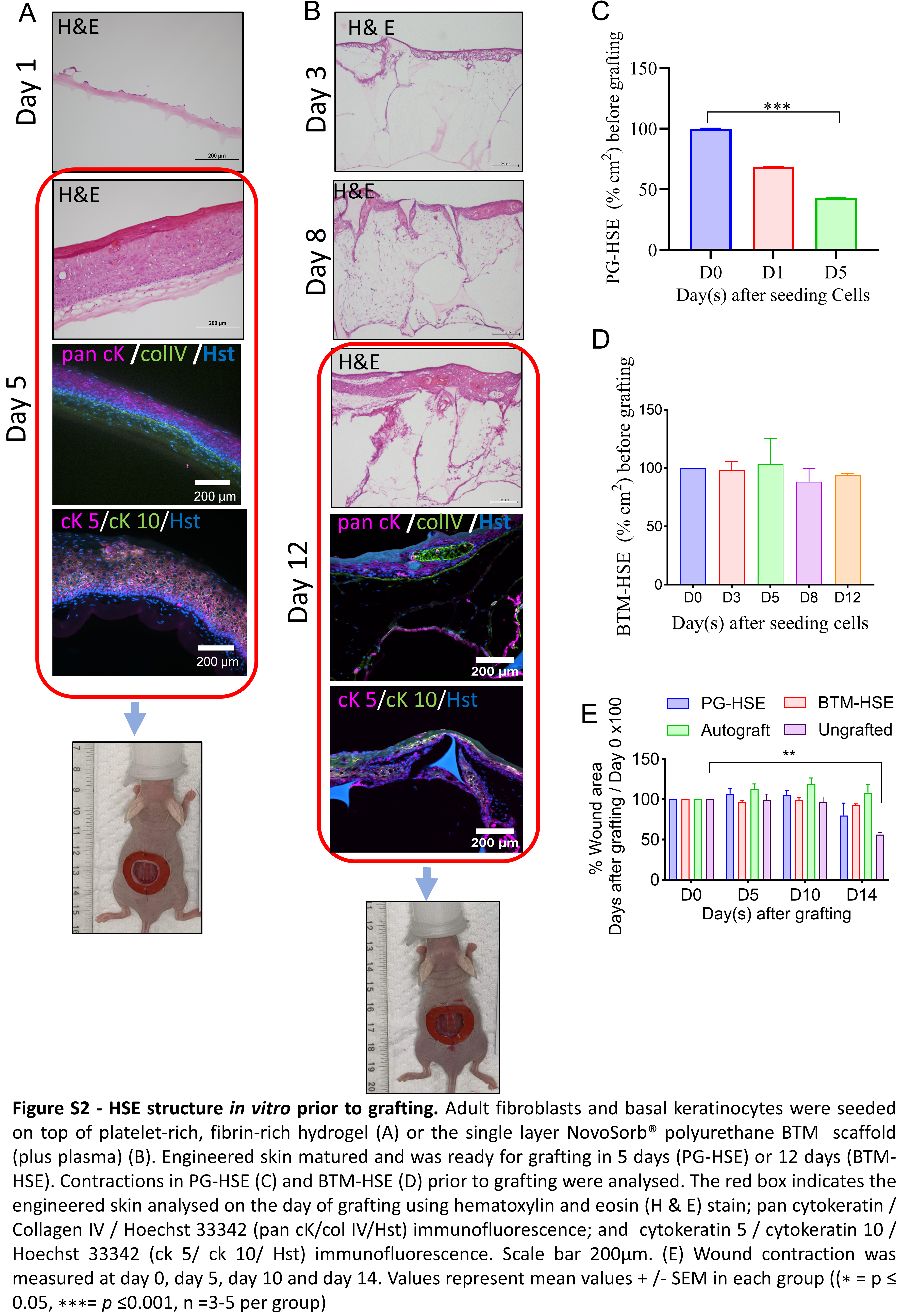

Supplement: Supplementary file 1 [file ijms-26-09988-s001.zip › Figure S2.TIF]

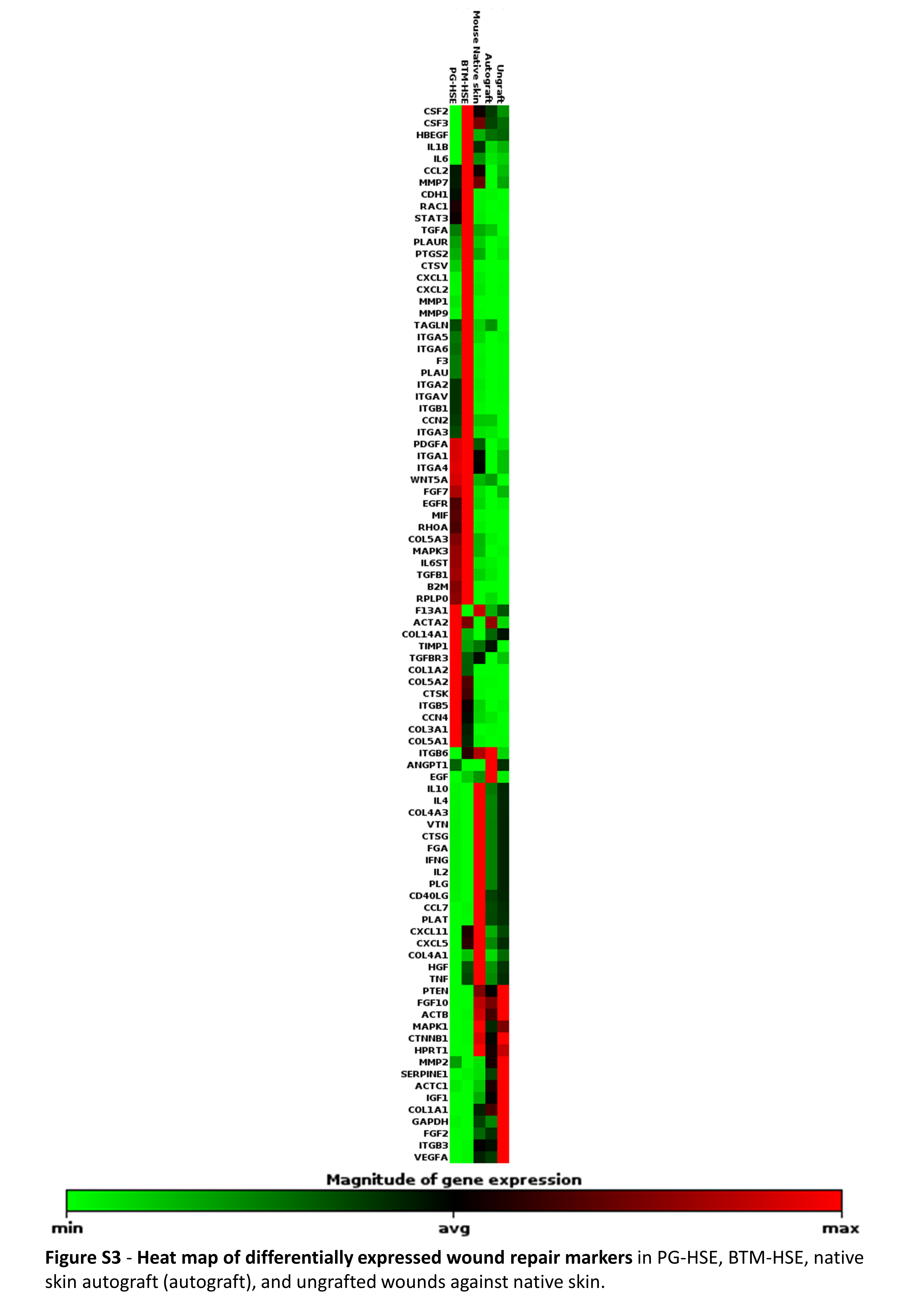

Supplement: Supplementary file 1 [file ijms-26-09988-s001.zip › Figure S3.TIF]

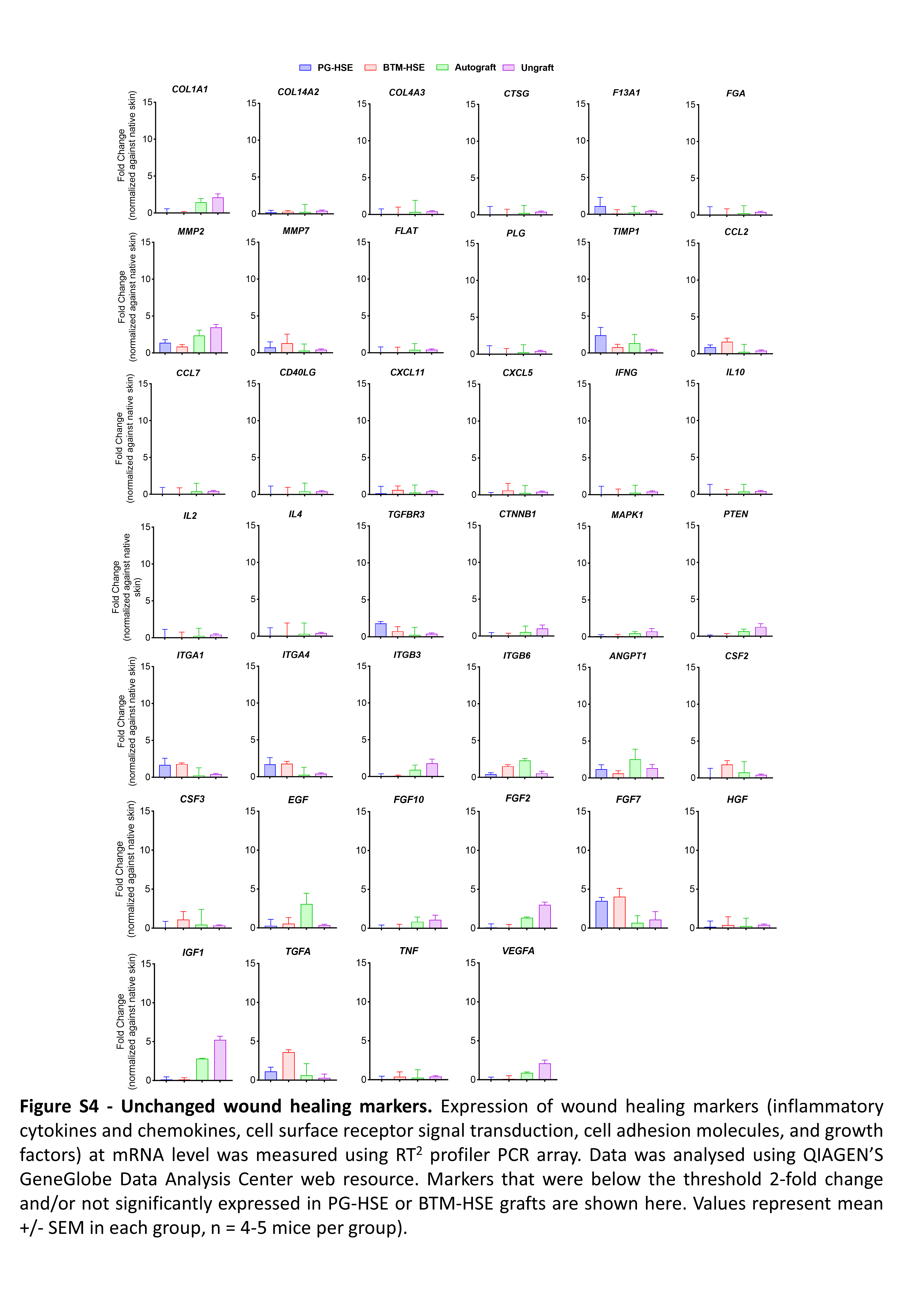

Supplement: Supplementary file 1 [file ijms-26-09988-s001.zip › Figure S4.TIF]

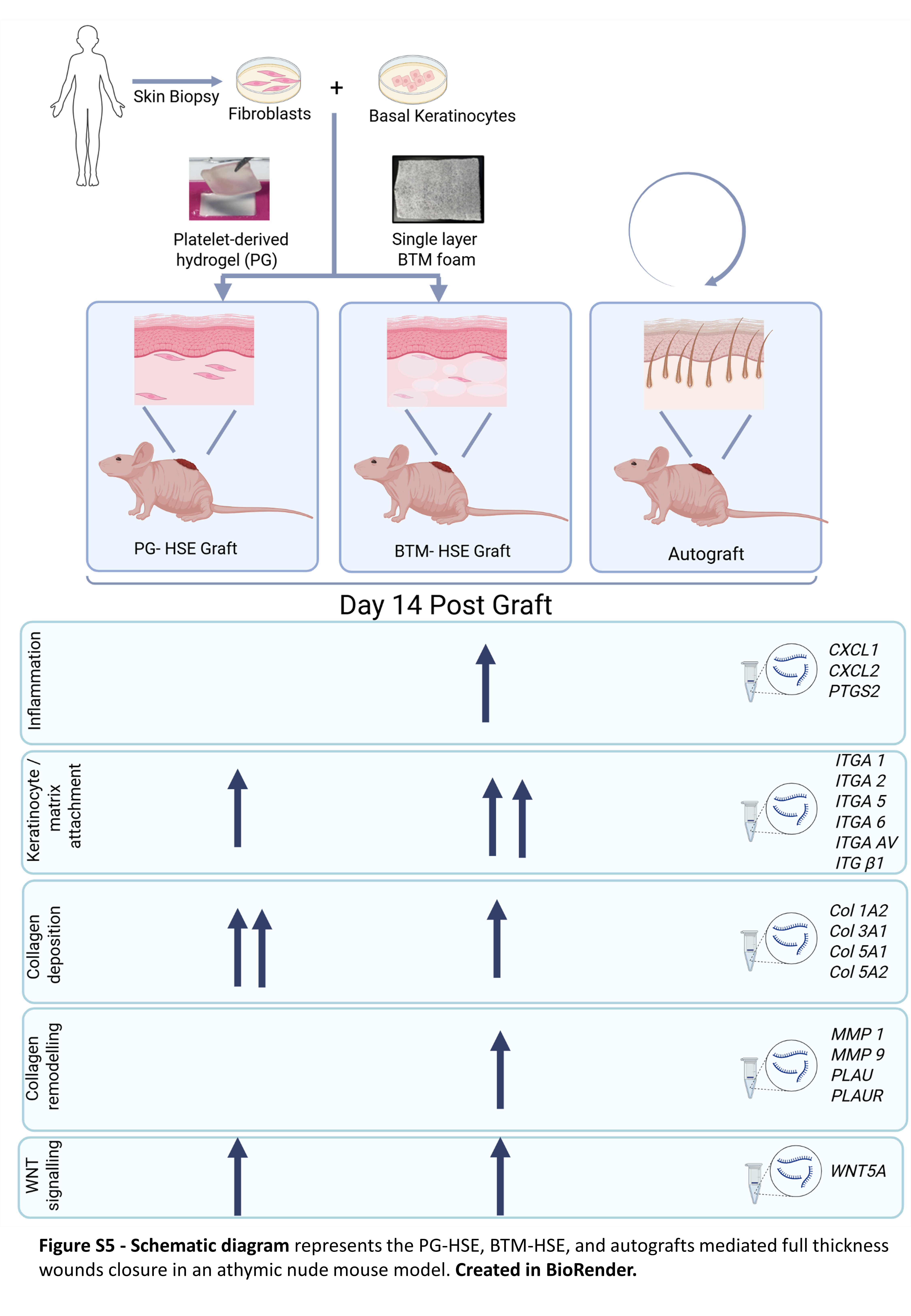

Supplement: Supplementary file 1 [file ijms-26-09988-s001.zip › Figure S5.TIF]
